# Intersphincteric abdominoperineal excision versus low Hartmann’s procedure in the robotic era: a single institution experience

**DOI:** 10.1007/s00423-026-04115-1

**Published:** 2026-07-02

**Authors:** Muthanna Al-Jumaili, Aous Al-Sudany, Karam Matlub, Leena Anna Marie Vanderberg, Mark Bremholm Ellebæk, Nuunu Nielsen, Patrick Fuhlendorf, Issam Al-Najami

**Affiliations:** 1 Department of Surgery, Odense University Hospital, Odense, Denmark; 2 Department of Clinical Research, University of Southern Denmark, Odense, Denmark; 3 Research Unit for Surgery, Odense University Hospital, University of Southern Denmark, Odense, Denmark

**Keywords:** Rectal cancer, Hartmann’s procedure, Intersphincteric abdominoperineal excision, Robotic-assisted surgery, Non-restorative rectal resection, Postoperative complications

## Abstract

**Purpose:**

Non-restorative surgical options for rectal cancer patients when primary anastomosis is not suitable, Hartmann’s procedure (HP) or intersphincteric abdominoperineal excision (IAPE) are common. Previous studies suggest higher pelvic sepsis after HP, but no data exist comparing exclusively robot-assisted approaches.

**Methods:**

This retrospective study included rectal cancer patients with significant comorbidities precluding anastomosis. Those patients underwent robot-assisted HP or IAPE at Odense University Hospital (2012–2022). Demographic, 30-day postoperative medical and surgical complications, reoperation, readmission, and 90-day mortality were analysed.

**Results:**

A total 224 patients were included (HP: *n* = 57; IAPE: *n* = 167), HP patients were older (77.0 ± 9.3 vs. 71.7 ± 11.5 years, *p* = 0.002) and had higher ASA scores (*p* = 0.001). Postoperative medical complications were low and comparable in both groups. Intra abdominal abscess was also comparable (3.5% for HP vs. 5.4% for IAPE, *p* = 0.832) Perineal wound infection still present in the IAPE group (6.6%) and perineal pain at 14 day was relatively high (11.4%) after IAPE. No significant difference was observed in the rate of readmission, reoperation and 90-day mortality.

**Conclusion:**

Complications rates did not significantly differ between HP and IAPE. However, IAPE was associated with perineal wound-related morbidity. In patents unfit for anastomosis, robot-assisted HP may considered a better and safer alternative for IAPE.

## Introduction

The standard surgical treatment for rectal cancer is radical resection with restoration of bowel continuity [1]. Patients diagnosed with rectal cancer are often elderly and frequently have multiple comorbidities [2]. Although the tumour may be surgically resectable and an anastomosis technically feasible, cardiovascular and pulmonary comorbidities significantly increase the risk of anastomotic leakage, leading to higher morbidity and mortality; therefore, anastomosis is often omitted in these patients [1, 3].

In patients deemed unsuitable for anastomosis, surgeons generally have two primary non-restorative surgical options when the tumour is resectable. Both procedures involve total mesorectal excision (TME) with dissection to the pelvic floor and result in a permanent end colostomy. These include a low Hartmann’s procedure (HP), in which the distal rectal remnant is stapled at the pelvic floor, and intersphincteric abdominoperineal excision (IAPE).

HP has traditionally been recommended for frail and elderly patients, as it is generally a shorter and technically less demanding procedure. However, it is associated with a risk of intra-abdominal infection and pelvic sepsis, which are reported as common postoperative complications [4, 5]. Alternatively, IAPE may be performed. Although it is a more extensive and time-consuming procedure, it is associated with a lower risk of pelvic abscess formation. However, perineal pain and perineal wound infections are frequently reported and may result in prolonged hospital stay and increased need for antibiotic treatment [6, 7].

In recent years, robotic-assisted surgery has increasingly been adopted to facilitate complex pelvic dissection in rectal cancer surgery. To date, no study has directly compared outcomes of HP and IAPE performed exclusively using robotic-assisted techniques.

This study aims to compare postoperative complications and associated risks between HP and IAPE in patients undergoing non-restorative surgery for rectal cancer, with both procedures performed exclusively using robot-assisted surgery.

## Materials and methods

### Study design

This retrospective cohort study was conducted at the Department of Surgery, Odense University Hospital, Denmark. All patients diagnosed with rectal cancer between 2012 and 2022 who were considered unsuitable for primary anastomosis due to significant comorbidities were included.

All patients underwent robot-assisted surgery and received either HP or IAPE. Allocation to procedure was based on clinical factors, including patient comorbidity, tumour height, age, and the operating surgeon’s assessment. Pathological reports were reviewed to confirm that all patients underwent total mesorectal excision with dissection to the pelvic floor. The study is reported in accordance with the STROBE (Strengthening the Reporting of Observational Studies in Epidemiology) guidelines for observational cohort studies [8].

## Procedures

All procedures where completed on davinci XI platform, utilizing 5 ports placement in the lower abdomen, when the rectum was transected a sureform black stapler 60 mm was used. The perineal wound was closed with a monofilament 4 − 0 running dermal suture. All procedures were performed by two senior surgeons, each with an expertise of over minimum 200 robotic colorectal cancer procedures.

## TME

All the specimen where complete, all specimen had free margins. The number of harvested lymph nodes where > 12 in all specimens, with no significant difference in lymph node harvest between the 2 groups.

## Data collection

Clinical and demographic data were retrieved from electronic medical records, including outpatient clinic notes, pre- and postoperative assessments, operative reports, postoperative follow-up notes, and pathology, CT, and MRI reports. Baseline variables included age, sex, smoking status, body mass index (BMI), American Society of Anesthesiologists (ASA) classification, World Health Organization (WHO) performance status, tumour stage, tumour height measured from the anal verge, and information on neoadjuvant and adjuvant chemotherapy or radiotherapy.

Perineal pain was assessed based on documentation from scheduled postoperative outpatient visits at 14 days and six weeks. At these visits, patients were asked whether they experienced abdominal or perineal pain, with responses recorded as yes or no. No visual analogue scale (VAS) or other pain scoring system was applied, and no standardized questionnaire was used for pain assessment.

## Outcomes

The primary outcomes were postoperative surgical and medical complications occurring within 30 days. Surgical complications included intra-abdominal abscess, bleeding, bowel injury, perineal wound infection, and rectal stump blowout. Secondary outcomes included reoperation rate, hospital readmission, total length of hospital stay, and 90-day postoperative mortality. Perineal pain was evaluated at outpatient follow-up visits 14 days and six weeks after discharge.

### Data storage

All study data were entered into REDCap (Research Electronic Data Capture), hosted by OPEN (Odense Patient Data Explorative Network, www.sdu.dk/open), and were additionally stored on the Department of Surgery’s secure SharePoint platform designated for this study.

## Ethics

The study was conducted in accordance with regional and institutional regulations for research involving human data. Approval was obtained from the Region of Southern Denmark Register of Research Projects (project ID: 22/40242) and the Regional Secretariat and Legal Office (project ID: 23/4152). According to Danish legislation, no further ethical approval was required, as the study was based exclusively on registry and clinical data without direct patient contact.

### Statistical analysis

Statistical analysis was performed to compare baseline characteristics between HP and IAPE. Continuous variables were reported as mean with standard deviation and compared using Student’s t-test. Categorical variables were reported as counts with percentages and compared using the chi-squared test. Fisher’s exact test was applied when expected cell counts were below 5. A two-sided p-value below 0.05 was considered statistically significant. Analyses were performed using R.

## Results

### Demographic data

**Fig. 1:**
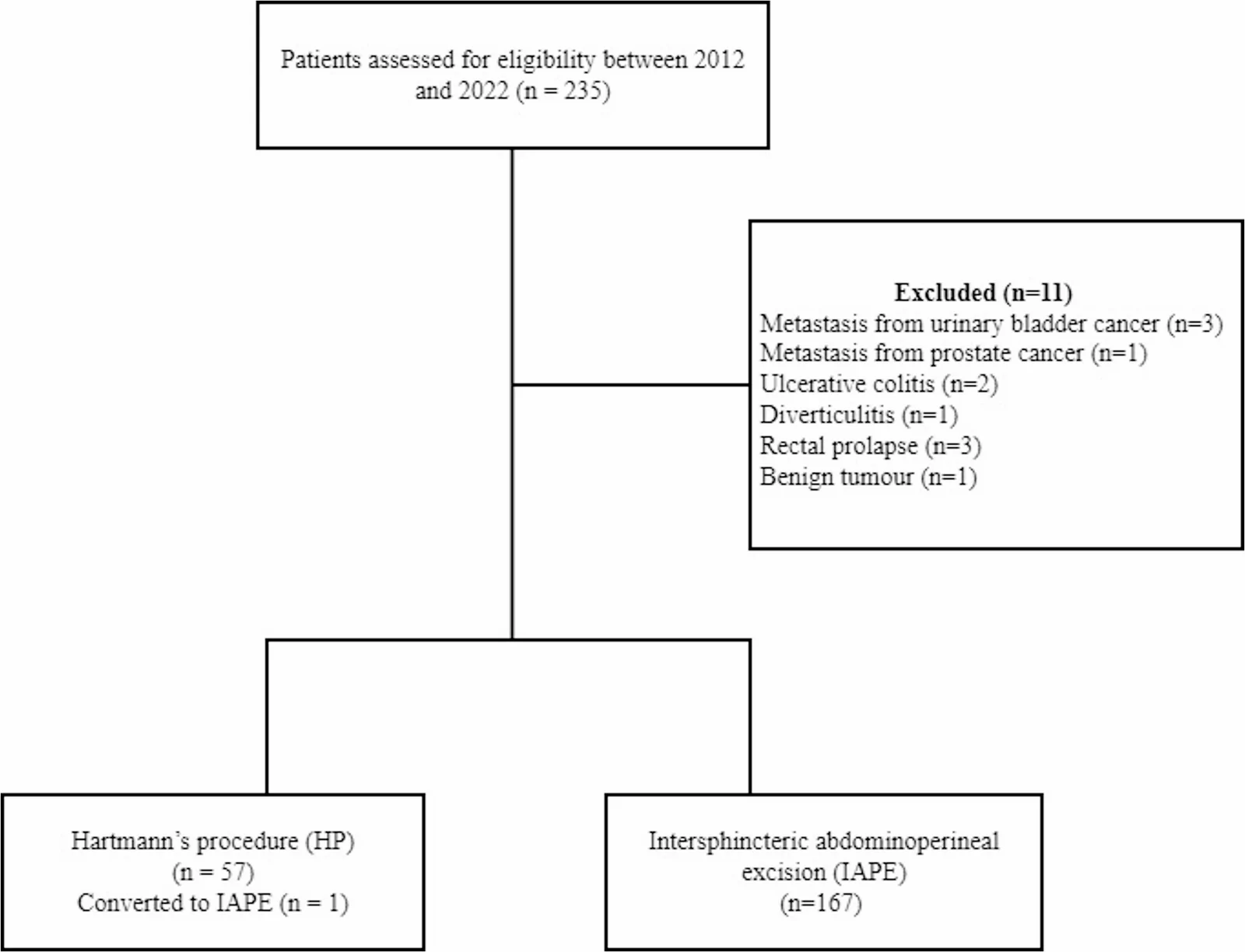
Study flowchart

Between 2012 and 2022, 235 patients underwent non-restorative surgery for rectal cancer. After exclusions, 224 patients were included: 57 underwent HP and 167 underwent IAPE (Fig. 1).

The mean age was significantly higher in the HP group than in the IAPE group (76.98 ± 9.27 vs. 71.65 ± 11.50 years; *p* = 0.002). Gender distribution, smoking status, and BMI were comparable between groups.

ASA score distribution differed significantly (*p* = 0.001). In the HP group, 47.4% were classified as ASA II, 47.4% as ASA III, and 3.5% as ASA IV. In contrast, the IAPE group included a higher proportion of ASA II patients (59.9%), fewer ASA III patients (26.9%), and no ASA IV patients (Table 1).

**Table 1:** Patients’ demographics in the HP and IAPE Groups

Variable		HP (*n* = 57)	IAPE (*n* = 167)	*p*-value
Age (mean (SD))		76.9 (9.2)	71.6 (11.5)	0.002
Gender, n (%)	Female	20 (35.1%)	58 (34.7%)	1.000
	Male	37 (64.9%)	109 (65.3%)	
Smoking, n (%)	Non-/Former smoker	43 (75.4%)	128 (76.6%)	0.608
	Current smoker	13 (22.8%)	32 (19.2%)	
	Unknown	1 (1.8%)	7 (4.2%)	
BMI (mean (SD))		26.6 (6.1)	26.3 (4.9)	0.726
ASA Score, n (%)	ASA 1	1 (1.8%)	21 (12.6%)	0.001
	ASA 2	27 (47.4%)	100 (59.9%)	
	ASA 3	27 (47.4%)	45 (26.9%)	
	ASA 4	2 (3.5%)	0 (0.0%)	
	ASA uninformed	0 (0.0%)	1 (0.6%)	

### Tumour characteristics and preoperative treatment

Tumour height from the anal verge was significantly greater in the HP group (mean 11.89 cm ± 2.49) than in the IAPE group (6.40 cm ± 2.78; *p* < 0.001).

Pathological T-stage distribution did not differ significantly between groups, with T3 tumours predominating in both (66.7% in HP and 53.6% in IAPE). N-stage and M-stage distributions were also comparable (Table 2).

**Table 2:** TNM staging, tumor height, and neoadjuvant/adjuvant treatment, in the HP and IAPE Groups

Variable		Hartmann (*n* = 57)	IAPE (*n* = 167)	*p*-value
T-stage Pathology (%)	T1	2 (3.5)	14 (8.4)	0.314
	T2	15 (26.3)	57 (34.3)	
	T3	38 (66.7)	89 (53.6)	
	T4	2 (3.5)	6 (3.6)	
N-stage Pathology (%)	N0	23 (40.4)	64 (38.6)	0.981
	N1	25 (43.9)	75 (45.2)	
	N2	8 (14.0)	25 (15.1)	
	Nx	1 (1.8)	2 (1.2)	
M-stage Pathology (%)	M0	50 (87.7)	148 (89.2)	0.957
	M1	7 (12.3)	18 (10.8)	
Tumor Height from anal verge (cm) (mean (SD))		11.8 (2.4)	6.4 (2.7)	< 0.001
chemo-radiotherapy (%)	No treatment	47 (82.5)	81 (48.5)	0.001
	Neoadjuvant chemotherapy	4 (7.0)	33 (19.8)	
	Neoadjuvant radiotherapy	1 (1.8)	27 (16.2)	
	Adjuvant chemotherapy	5 (8.8)	23 (13.8)	
	Adjuvant radiotherapy	0 (0.0)	2 (1.2)	
	Adjuvant immune therapy	0 (0.0)	1 (0.6)	

A significantly higher proportion of patients in the HP group received no neoadjuvant chemoradiotherapy compared with the IAPE group (82.5% vs. 48.5%; *p* = 0.001). Neoadjuvant chemotherapy and radiotherapy were more frequently administered in the IAPE group, as was adjuvant chemotherapy (Table 2).

### Postoperative complications

Reoperation rates were similar between groups (12.3% HP vs. 13.2% IAPE; *p* = 1.000). Pelvic abscess formation occurred in 3.5% of HP patients and 5.4% of IAPE patients, with no significant difference (*p* = 0.832). Postoperative medical complications were infrequent in both groups (3.5% HP vs. 6.6% IAPE; *p* = 0.596) (Table 3).

**Table 3:** Post-operative medical complications, Length og stay, re-admision, re- operation and post-operative surgical complications within 30 days. Mortality within 90 days in the HP and IAPE Groups

Variable	Clavien-Dindo	Hartmann	IAPE
n		57	167
Post-operative medical complications (%)	-	2 (3.5)	11 (6.6)
Stroke (%)	2	1 (1.8)	1 (0.6)
Acute myocardial infarction (%)	-	0 (0.0)	0 (0.0)
Pneumonia (%)	1	0 (0.0)	2 (1.2)
	2	1 (1.8)	5 (3.0)
Heart failure (%)	0	0 (0.0)	0 (0.0)
Pulmonary embolism (%)	2	0 (0.0)	1 (0.6)
Respiratory failure (%)	4a	0 (0.0)	1 (0.6)
Renal failure (%)	4a	0 (0.0)	2 (1.2)
Sepsis (%)	2	0 (0.0)	1 (0.6)
	4a	0 (0.0)	1 (0.6)
DVT (%)	-	0 (0.0)	0 (0.0)
Length of stay (day) (mean (SD))	-	7.5 (9.7)	7.9 (7.5)
Re-admission within 30 days (%)	-	4 (7.0)	19 (11.4)
Re-operation within 30 days (%)	-	7 (12.3)	22 (13.2)
Ischemic colon (%)	-	1 (1.8)	1 (0.6)
Wound dehiscence (%)	-	2 (3.6)	1 (0.6)
Iatrogenic vaginal perforation (%)	-	0 (0.0)	1 (0.6)
Bowel obstruction (%)	-	2 (3.5)	8 (4.8)
Pelvic abscess (%)	-	0 (0.0)	1 (0.6)
Parastomal hernia (%)	-	0 (0.0)	1 (0.6)
Port site hernia (%)	-	1 (1.8)	0 (0.0)
Stoma revision (%)	-	0 (0.0)	6 (3.6)
Bowel injury (%)	-	0 (0.0)	3 (1.8)
Bleeding (%)	-	3 (5.3)	1 (0.6)
Perineal wound infection (%)	-	0 (0.0)	1 (0.6)
Leakage from rectal stump (%)	-	1 (1.8)	0 (0.0)
Pelvic abscess (%)	-	2 (3.5)	9 (5.4)
Perineal wound infection (%)	-	0 (0.0)	11 (6.6)
Perineal pain 14 days post-operative (%) *	-	0 (0.0)	19 (11.4)
Perineal pain 6 weeks post-operative (%)	-	1 (1.8)	8 (4.8)
Perineal hernia (%)	-	0 (0.0)	0 (0.0)
Mortality (90 days) (%)	-	0 (0.0)	4 (2.4)

*A significant difference was only observed in perineal pain 14 days postoperatively (*p* = 0.017)

Perineal wound infection occurred in 6.6% of patients in the IAPE group. Perineal pain at 14 days postoperatively was reported by 11.4% of IAPE patients, with 4.8% reporting persistent pain at six weeks.

Two patients in the HP group experienced rectal stump blowout; one required conversion to IAPE. 90-day mortality was low and comparable between groups (0% HP vs. 2.4% IAPE; *p* = 0.549).

## Discussion

Several studies have previously reported an increased incidence of pelvic abscess and blow-out of the rectal stump following HP compared to IAPE [4, 5, 9, 10].

However, our findings do not support this trend in our population of patients operated robotically only, as the rates of these complications were found to be comparable between the two groups.

Specifically, the incidence of abscess formation did not differ significantly. These were 3.5% efter HP vs. 5.4% after IAPE (*p* = 0.832). This incidence is relatively lower compared to findings from other studies in which robotic surgery was not employed [3–5, 9–11].

In a large prospective study, the overall rate of surgical complications was similar between the two procedures (51% for HP vs. 48% for IAPE, *p* = 0.737), with pelvic abscess occurring in 15% of patients undergoing HP, compared with only 1% in IAPE group [3]. Another retrospective study also reported comparable rates of postoperative complications (21% for HP vs. 26% for IAPE), including wound infections (6% for HP vs. 12% for IAPE) and intra-abdominal infections (6% for HP vs. 5% for IAPE) [11].

In our study, postoperative medical complications were relatively low in both groups, suggesting overall acceptable short-term outcomes regardless of the procedure performed.

Despite these similarities, certain procedure-specific complications persisted. Notably, postoperative perineal wound infection and perineal pain were still observed among patients in the IAPE group. These complications may be influenced by multiple factors, including surgical approach and extent of pelvic dissection.

It is essential to interpret these outcomes in the context of baseline differences between the two patient groups. Notably, the distribution of ASA-scores differed significantly, with patients in the HP group more often presenting with higher scores, reflecting greater preoperative comorbidity. Furthermore, the mean age was significantly higher in the HP group. Both factors are known to influence postoperative outcomes and should be considered when evaluating outcomes.

Conversely, tumor height was significantly lower in the IAPE group, which typically necessitates more extensive pelvic dissection and technically more challenging operative conditions. Although we tried to mitigate the differences by including TME only procedures making the pelvic dissection more comparable. This, in combination with the more frequent use of neoadjuvant and adjuvant chemo-radiotherapy in the IAPE group, may negatively affect tissue healing and increase the risk of postoperative complications.

Several limitations of this study should be acknowledged. First, its retrospective design introduces inherent selection bias, as the choice of surgical procedure was largely based on the surgeon’s preference, influenced by patient condition and intraoperative findings, rather than randomized allocation. Second, the assessment of postoperative perineal pain was based on information collected during follow-up visits at 14 days and six weeks. Although no patients dropped out during the follow-up period, these visits were not specifically designed to evaluate this symptom. As a result, the actual incidence of perineal pain may have been underreported by patients or under documented by clinicians. Ideally, patient-reported outcomes should have been collected to provide a more comprehensive understanding of the procedure’s impact on patients’ quality of life, not limited to pain alone.

To date, as far as we know, no studies have investigated the differences between the two methods when performed exclusively with robotic assistance. The HAPIrect trial directly compared HP and IAPE as treatment options for rectal cancer located 5 cm or more from the anal verge in patients with comorbidities that increase the risk associated with low anterior resection and anastomosis. In this study, both laparoscopic and robotic assisted surgery, even open surgery was included [2]. The recently published results of this RCT revealed no statistically significant difference in the overall rate of postoperative surgical complications within 30 days between the two groups. However, pelvic abscesses were observed more frequently in the HP group, affecting approximately 9% of patients, compared with 2.4% in the IAPE group [12].

## Conclusion

The increasing use of robotic-assisted surgery in non-restorative rectal cancer surgery appears to be associated with favourable short-term outcomes and low rates of postoperative complications. In our cohort, a higher incidence of perineal pain and wound infection was observed following IAPE, alongside longer operative times. While these results are likely influenced by the lower tumor height and higher rates of neoadjuvant therapy in the IAPE group, they highlight the technical challenges and increased morbidity associated with this approach in a real-world setting. Further prospective randomized studies focusing on robotic HP versus robotic IAPE, including patient-reported outcomes, are warranted to better define the optimal surgical strategy for this patient population.

## Data Availability

The data supporting the findings of this study are available from the corresponding author upon reasonable request and in accordance with institutional and regional data protection regulations.
